# Online yoga in myeloproliferative neoplasm patients: results of a randomized pilot trial to inform future research

**DOI:** 10.1186/s12906-019-2530-8

**Published:** 2019-06-07

**Authors:** Jennifer Huberty, Ryan Eckert, Amylou Dueck, Heidi Kosiorek, Linda Larkey, Krisstina Gowin, Ruben Mesa

**Affiliations:** 1 Exercise Science and Health Promotion, Arizona State University, 500 North 3rd Street Phoenix, Tempe, AZ 85004 USA; 2 Mays Cancer Center, University of Texas San Antonio MD Anderson, 7979 Wurzbach Road, San Antonio, TX 78229 USA; 3 Division of Biostatistics, Mayo Clinic, 13400 East Shea Boulevard, Scottsdale, AZ 85259 USA; 4 College of Nursing and Health Innovation, Arizona State University, 550 North 3rd Street, Phoenix, AZ 85004 USA; 5 University of Arizona, 1501 North Campbell Avenue, Tucson, AZ 85724 USA

**Keywords:** Cancer, Mindfulness, Complementary, Physical activity, Neoplasm

## Abstract

**Background:**

Myeloproliferative neoplasm (MPN) patients suffer from significant symptoms, inflammation and reduced quality of life. Yoga improves these outcomes in other cancers, but this hasn’t been demonstrated in MPNs. The purpose of this study was to: (1) explore the limited efficacy (does the program show promise of success) of a 12-week online yoga intervention among MPN patients on symptom burden and quality of life and (2) determine feasibility (practicality: to what extent a measure can be carried out) of remotely collecting inflammatory biomarkers.

**Methods:**

Patients were recruited nationally and randomized to online yoga (60 min/week of yoga) or wait-list control (asked to maintain normal activity). Weekly yoga minutes were collected with Clicky (online web analytics tool) and self-report. Those in online yoga completed a blood draw at baseline and week 12 to assess inflammation (interleukin-6, tumor necrosis factor-*alpha* [TNF-α]). All participants completed questionnaires assessing depression, anxiety, fatigue, pain, sleep disturbance, sexual function, total symptom burden, global health, and quality of life at baseline, week seven, 12, and 16. Change from baseline at each time point was computed by group and effect sizes were calculated. Pre-post intervention change in inflammation for the yoga group was compared by t-test.

**Results:**

Sixty-two MPN patients enrolled and 48 completed the intervention (online yoga = 27; control group = 21). Yoga participation averaged 40.8 min/week via Clicky and 56.1 min/week via self-report. Small/moderate effect sizes were generated from the yoga intervention for sleep disturbance (d = − 0.26 to − 0.61), pain intensity (d = − 0.34 to − 0.51), anxiety (d = − 0.27 to − 0.37), and depression (d = − 0.53 to − 0.78). A total of 92.6 and 70.4% of online yoga participants completed the blood draw at baseline and week 12, respectively, and there was a decrease in TNF-α from baseline to week 12 (− 1.3 ± 1.5 pg/ml).

**Conclusions:**

Online yoga demonstrated small effects on sleep, pain, and anxiety as well as a moderate effect on depression. Remote blood draw procedures are feasible and the effect size of the intervention on TNF-α was large. Future fully powered randomized controlled trials are needed to test for efficacy.

**Trial registration:**

This trial was retrospectively registered with clinicaltrials.gov (ID: NCT03503838) on 4/19/2018.

## Background

Myeloproliferative neoplasms (MPNs), including polycythemia vera, essential thrombocytosis, and myelofibrosis are a rare hematological cancers characterized by overproduction of blood cells, enlarged spleen, risk of thrombotic and hemorrhagic events, and risk of transformation to acute myeloid leukemia. Over the preceding decade, MPN symptom burden has been emerging as a key therapeutic treatment target for this population. The MPN Symptom Assessment Form (MPN-SAF) [1–3] has been developed and validated as a clinical tool to assess this multifactor symptom profile reflecting the therapeutic target.

With the discovery of one of the key mechanisms of the disease, JAK V617F driver mutations, in 2005 [4], JAK inhibitor therapy has drastically changed the landscape of treatment for MPNs leading to improvements in spleen enlargement, symptom burden, and overall survival [5, 6]. With very limited options for curative intervention (e.g., allogeneic stem cell transplant only available for a very small percentage of patients) and incomplete symptom resolution, MPN is for most a chronic, lifelong disease. Fatigue, one of the most common and debilitating symptoms in MPN patients, is reported in upwards of 93% of MPN patients, and is thought to be the largest contributor to reduced quality of life [2, 7]. Additionally, disease-related psychosocial sequelae, such as depression, are often refractory and undertreated [8, 9].

In recent years, research has demonstrated the physical and psychosocial benefits of yoga in cancer patients, particularly breast cancer [10–13]. Multiple systematic reviews and meta-analyses have found that yoga is efficacious for improving a range of outcomes in cancer patients, including improvements in functional well-being, distress, anxiety, depression, fatigue, emotional function, social function, sleep quality, and overall quality of life [10–13]. Additionally, yoga is reported as one of the most popular complementary and alternative medicine approaches utilized by cancer survivors in the United States [14]. Yoga, could be an effective strategy to reduce MPN patient symptom burden that is often left unaddressed with current pharmacologic therapy.

In a survey of over 850 MPN patients, cross-sectional data demonstrated that 26% of MPN patients (*n* = 220) reported participating in yoga and that self-reported yoga participation was significantly associated with an improved total symptom burden (*p* = 0.02), reduced depressive symptoms (*p* = 0.001), and improved quality of life (p = 0.02) [15]. Furthermore, a recent study by Huberty et al. [16] explored a 12-week online yoga (OLY) intervention in MPN patients (*n* = 38) and found yoga to be feasible (i.e., acceptable & practical), with average weekly self-reported yoga participation of ~ 50 min/week (60 min/week was prescribed) and a high rate of satisfaction (i.e., 68% satisfied or very satisfied and 75% found it helpful for coping with symptoms). From baseline to post-intervention (i.e., week 12) with no comparison group, MPN patients reported significantly improved total symptom burden (ES = -0.36; *p* = 0.004), anxiety (ES = -0.67; *p* = 0.002), depression (ES = -0.41; *p* = 0.049), sleep disturbance (ES = -0.58; *p* < 0.001), and fatigue (ES = -0.33; p = 0.04), with no significant differences in outcomes between those that averaged ≥60 min/week of yoga and those that averaged < 60 min/week of yoga [16].

Prior research demonstrates that specific pro-inflammatory cytokines (e.g., interleukin [IL]-1, IL-6, IL-8, and tumor necrosis factor-α [TNF-α]) are associated with worsened patient-reported symptoms, including fatigue, abdominal complaints, microvascular symptoms, and constitutional symptoms [17]. Although yoga therapy studies among solid tumor cancer patients have consistently shown response to these same inflammatory markers, MPN patients’ inflammation is markedly worse, and is intrinsic to the disease process itself.

The purpose of this study was to: (1) explore the limited efficacy (i.e., does the program show promise of being successful with the intendend population) of a 12-week online yoga intervention among MPN patients on symptom burden and quality of life and (2) determine feasibility (i.e., practicality; to what extent a measure can be carried out with intendend participants using existing means) of remotely collecting inflammatory biomarkers. The preliminary data gathered from this study will inform the design of a future randomized controlled trial to examine efficacy.

## Methods

MPN patients (*n* = 60) were recruited online through MPN organizational partners. MPN patients interested in the study were asked to complete an online eligibility questionnaire administered via Qualtrics (Provo, UT). Researchers then checked eligibility questionnaires as they were completed and emailed interested patients with their eligibility status. MPN patients were eligible if they a) had a diagnosis of essential thrombocythemia, polycythemia vera, or myelofibrosis, b) answered “no” to all items on the Physical Activity Readiness Questionnaire [18] or were willing to obtain a signed medical release, c) had access to a desktop/laptop on a regular basis, d) had access to reliable internet, e) were able to read/understand English, f) were ≥ 18 years of age, g) were willing to be randomized to OLY or CG, and h) were willing to drive to nearest Quest Diagnostics Patient Service Center for a blood draw. MPN patients were ineligible if they a) reported performing tai chi, qi gong, or yoga for ≥60 min/week, b) reported engaging in ≥150 min/week of physical activity, c) utilized the online yoga site to be used for the study, Udaya.com, d) reported a history of syncope in the last two months, e) reported a history of recurrent falls (≥2 in past two months), f) had a score of ≥15 on the PHQ-9, g) reported a score of > 3 on the ECOG-3, h) were pregnant, or i) resided outside of the US.

If ineligible, patients received an email stating their ineligibility status, a discount code for a Udaya.com membership and instructions for accessing the online yoga prescription. MPN patients who were eligible were asked to set up a 20-min phone intake appointment in which the study details and informed consent was described in detail. MPN patients who completed the intake appointment were then sent an informed consent electronically via Qualtrics (Provo, UT) that included a place for their electronic signature. Upon receipt of the completed informed consent, participants were randomized to either OLY or CG. A group assignment list was generated by a research assistant prior to study commencement with randomizer.org. This pre-generated list was then used by a different research assistant to assign eligible and consented MPN patients into a study arm in the order in which they consented to participate. Staff were not involved in data collection (rather, participant data was entered directly electronically) and thus blinding staff to study condition was not a threat to validity. Participants were not blinded to randomization assignment; either receiving the yoga intervention or understanding that they were to continue normal activity.

### Yoga group

Participants randomized to the OLY group were asked to complete 60 min/week of home-based, online-streamed yoga via Udaya.com for 12 weeks. Participants were asked to complete 60 min of yoga each week. Investigators (physician specializing in MPNs, PhD trained researcher/certified yoga instructor, MS trained biomechanist/yoga educator) developed a prescription selected from a video library (Udaya.com) of progressively mild- to moderate-intensity yoga classes based on Hatha and Vinyasa-style classes. All videos met the following criteria: 1) rated as either “beginner” or “intermediate” and 2) excluded poses requiring the participant to lay on their stomach (out of concern for an enlarged spleen or liver). All yoga classes on Udaya are taught by trained yoga instructors (> 500 h registered yoga instructor). In partnership with Udaya.com, the research team also filmed a total of six yoga videos specifically for the purpose of this study. The filmed classes were all taught by a yoga biomechanist/instructor using the same criteria above.

Weeks 1–2 were based on brief introductory videos (5–20 min) demonstrating fundamental yoga poses and safe practice guidelines. Weeks 3–12 gradually increased to longer duration videos (20–30 min) with slightly increased intensity. All Udaya.com videos include a proper warm-up and cool down, reminders for breathing with the movements/poses, and a closing mindfulness activity with a message from yoga instructor, brief meditation, and final relaxation. Each week the yoga prescription totaled approximately 60 min. However, if participants wished to do more than the prescribed 60 min of yoga, participants could select a yoga class from the additional videos provided each week. The yoga prescription also included meditation-based yoga videos as additional videos each week based on study participant feedback from our prior feasibility study [16].

### Wait-list control group

CG participants were asked to maintain their usual activity for 16 weeks (i.e., baseline through to follow-up) prior to being offered OLY. CG participants were not asked to complete the blood draws, daily yoga logs, or any measures beyond the 16-week study period. At 16 weeks, CG participants were provided the same online prescription and materials that the OLY group received.

### Outcome assessments

All outcomes below were pre-defined and there were no changes to any outcomes after trial commencement.

#### Yoga participation/ safety

OLY participation was measured by number of minutes spent viewing yoga videos using Clicky (i.e., web analytics program) and self-reported by weekly logs.

Adverse events were both actively and passively recorded by research staff. Adverse events reported by study participants via email or phone were recorded in the case that this occurred. Participants also completed a daily log that asked “if they experienced any pain or discomfort performing the poses” each week.

#### Blood draws

Participants in the OLY group were also asked to obtain a blood draw at a nearby Quest Diagnostics Patient Service Center at baseline and week 12. Incentives included $10/$20 for completing baseline/post-intervention blood draw, respectively. Feasibility of obtaining remote blood draws was assessed according to guidelines outlined by Bowen et al. [19] for practicality. A priori blood draw practicality was defined as ≥70% completion rate at both baseline and post-intervention. Blood draws measured specific serum cytokines (i.e., IL-6, TNF-*a*).

#### Patient-reported outcomes

Patient-reported outcomes were assessed in all study participants electronically via a Qualtrics (Provo, UT) questionnaire at baseline, mid-point, post-intervention, and follow-up. The multifactor MPN Symptom Assessment Form (MPN-SAF) and National Institutes of Health Patient Reported Outcomes Measurement Information System (NIH PROMIS) scales were used. MPN-SAF measures included total symptom score (MPN-SAF TSS) as well as single-item MPN-SAF fatigue (question #1 on MPN-SAF). NIH PROMIS measures included Pain Intensity Short Form 3a (3-item), Emotional Distress-Anxiety Short Form 8a (8-item), Emotional Distress-Depression Short Form 8a (8-item), Sleep Disturbance Short Form 8a (8-item), Sexual Function (8-item for males; 10-item for females), Global-10 (10-item), and quality of life (i.e., question #2 on Global-10 scale). These scales are all reliable and valid for use in cancer patients [20–22]. Minimally important differences on NIH PROMIS measures have been defined as one-half standard deviation (5 points) [23].

### Statistical analysis

Self-reported yoga minutes based on weekly logs were compared (using a +/− 5 min buffer) to Clicky minutes by t-test. For patient-reported outcomes (MPN-SAF, NIH PROMIS) and laboratory values (i.e., blood biomarkers), change from baseline for each domain at each time point was computed by group. Effect size (ES) was calculated as the difference in means between OLY and CG divided by the pooled SD and interpreted according to Cohen’s d (0.2 = small, 0.5 = moderate, 0.8 = large) [24]. SAS version 9.4 (SAS Institute, Cary, NC) was used for analysis.

## Results

**Fig. 1:**
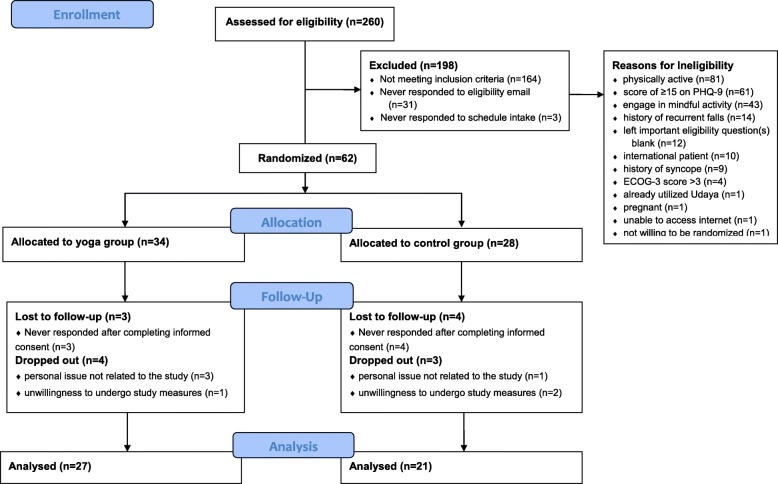
A depiction of study participant enrollment into the study

A total of 260 MPN patients were recruited and completed the eligibility survey between September 23, 2016 and February 28, 2017 (i.e., five months). The eligibility survey for this study closed after receiving 260 responses as the enrollment of 60 MPN patients had been met. Of the 260 MPN patients who completed the eligibility survey, 37% (*n* = 96) were eligible. Eligible participants were enrolled and randomized into the study in the order they completed the eligibility questionnaire. Sixty-two patients were enrolled, with 34 allocated to OLY and 28 allocated to CG. Of those randomized to OLY, 27 (79.4%) completed the study. Of those randomized to CG, 21 (75%) completed the data collection up through follow-up. There were no adverse events that were reported in either the OLY of CG during the course of the study. Data analysis was conducted on those that completed the intervention (i.e., completed data collection through follow-up). See Fig. 1 for study participant enrollment.

### Patient demographics

The most common diagnosis was essential thrombocythemia (*n* = 19), followed by polycythemia vera (*n* = 15) and myelofibrosis (*n* = 14). Participants were primarily female (*n* = 45), Caucasian (n = 45), married (*n* = 41), and had a Bachelor’s degree education or higher (*n* = 40). Most participants were diagnosed with MPN more than three years ago (*n* = 33). Furthermore, 47.9% (*n* = 23) indicated a history of anemia and 35.4% (*n* = 17) reported enlarged spleen. Only 20.8% (*n* = 10) of participants were being treated with Ruxolitinib or another JAK-Inhibitor. See Table 1 for study participant baseline demographics. *(insert* Table 1 *about here).*

**Table 1:** Baseline Demographics & Patient-Reported Outcomes

	Yoga Group (*n* = 27)	Control Group (*n* = 21)	Total (*n* = 48)	
	N (%)	N (%)	N (%)	p
Age, years (M ± SD)	58.3 ± 9.3	55.0 ± 11.4	56.9 ± 10.3	0.28
Body Mass Index (M ± SD)	26.6 ± 5.4	26.2 ± 5.6	26.5 ± 5.4	0.81
Gender				0.71
Male	2 (7.4)	1 (4.8)	3 (6.3)	
Female	25 (92.6)	20 (95.2)	45 (93.8)	
Race				0.42
Caucasian	25 (92.6)	20 (95.2)	45 (93.8)	
Other	2 (7.4)	1 (4.8)	3 (6.3)	
Diagnosis				0.53
Polycythemia Vera	10 (37.0)	5 (23.8)	15 (31.3)	
Essential Thrombocythemia	9 (33.3)	10 (47.6)	19 (39.6)	
Myelofibrosis	8 (29.6)	6 (28.6)	14 (29.2)	
Time Since Diagnosis				0.44
< 1 year ago	3 (11.1)	4 (19.0)	7 (14.6)	
1–3 years ago	4 (14.8)	4 (19.0)	8 (16.7)	
> 3 years ago	20 (74.1)	13 (61.9)	33 (68.8)	
Presence of Enlarged Spleen				0.45
Yes	8 (29.6)	9 (42.9)	17 (35.4)	
No	17 (63.0)	12 (57.1)	29 (60.4)	
Missing	2 (7.4)	0 (0.0)	2 (4.2)	
History of Anemia				0.23
Yes	15 (55.6)	8 (38.1)	23 (47.9)	
No	12 (44.4)	13 (61.9)	25 (52.1)	
Ruxolitinib/Other Janus Kinase-Inhibitor Treatment				0.65
Yes	5 (18.5)	5 (23.8)	10 (20.8)	
No	22 (81.5)	16 (76.2)	38 (79.2)	
Education				0.3
< High School	0 (0.0)	0 (0.0)	0 (0.0)	
High school diploma	2 (7.4)	0 (0.0)	2 (4.2)	
Some college	1 (3.7)	2 (9.5)	3 (6.3)	
Associates/2-year degree	3 (11.1)	0 (0.0	3 (6.3)	
Bachelors degree	10 (37.0)	8 (38.1)	18 (37.5)	
Graduate school or above	11 (40.7)	11 (52.4)	22 (45.8)	
Marital status				0.28
Single	2 (7.4)	0 (0.0)	2 (4.2)	
Partnered/in a relationship	1 (3.7)	2 (9.5)	3 (6.3)	
Married	22 (81.5)	19 (90.5)	41 (85.4)	
Separated	2 (7.4)	0 (0.0)	2 (4.2)	
Divorced	0 (0.0)	0 (0.0)	0 (0.0)	

### Yoga participation

**Fig. 2:**
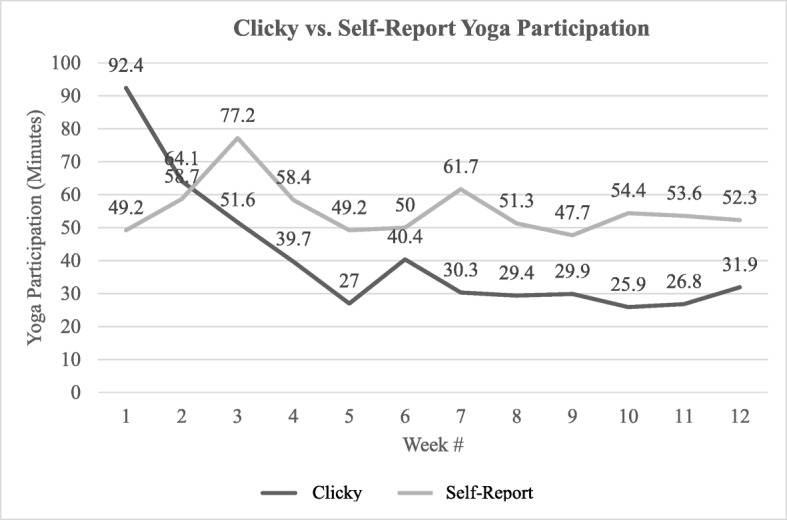
A comparison between self-report and Clicky yoga participation minutes

Yoga participation assessed via Clicky averaged 40.8 min/week with 15% (n = 4/27) averaging ≥60 min/week. There was a decrease in weekly yoga participation from ~ 92 min/week in week one to ~ 30 min/week in week seven. Weekly yoga participation remained relatively stable thereafter at around 25–30 min/week. Comparatively, self-report yoga participation averaged 56.1 min/week with 48% (*n* = 13/27) averaging ≥60 min/week. Weekly yoga participation increased during the first three weeks from ~ 49 min in week one to ~ 77 min in week three and then declined to ~ 49 min in week five and remained relatively stable thereafter at ~ 50–60 min/week. Figure 2 describes the weekly trend in both Clicky and self-report yoga participation.

Those in the OLY under-reported yoga participation in week one (− 45.5 ± 55.9 min). Alternatively, OLY participants over-reported yoga participation in weeks three through twelve by 10.0 ± 29.1 min at the lowest (week six) to 32.6 ± 38.9 min at the highest (week seven).

### Blood draw feasibility/practicality

**Fig. 3:**
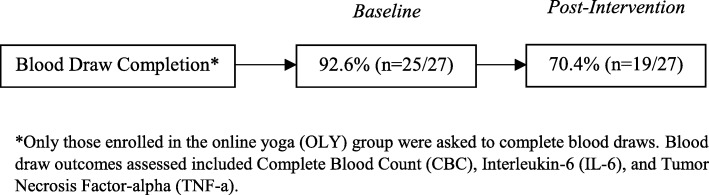
A visual depiction of blood draw completion rates

Average distance traveled for blood draw was 16.3 miles (range: 1.2–91.6 miles). No participants commented on the distance they had to travel to receive their blood draw. At baseline, 92.6% (*n* = 25/27) of OLY participants completed the blood draw and 70.4% (*n* = 19/27) completed the blood draw at post-intervention. See Fig. 3 for a depiction of blood draw completion rates.

### Patient-reported outcomes

There were small effects of the yoga intervention observed for sleep disturbances (d = − 0.26 to − 0.61), pain intensity (d = − 0.34 to − 0.51) and anxiety (d = − 0.27 to − 0.37). There was a moderate effect of the yoga intervention observed for depression (d = − 0.53 to − 0.78) Table 2 describes the changes in patient-reported outcomes by group and observed effect sizes for all domains. Minimally important differences (~ 5 points) were observed for physical and mental health measures seen in both yoga and control groups. *(insert* Table 2 *about here).*

**Table 2:** Baseline and changes from baseline scores for Patient-Reported Outcomes by group

		Yoga Group (*n* = 27)	Control Group (*n* = 21)	
		Mean (SD)	Mean (SD)	*d* ^*a*^
NIH Patient Reported Outcomes Measurement Information System Outcomes	Anxiety			
	Baseline	54.0 (7.8)	50.2 (8.6)	–
	Change from baseline, wk. 7	−2.1 (5.0)	−0.7 (5.4)	− 0.27
	Change from baseline, wk. 12	−3.1 (5.7)	−1.3 (7.1)	−0.30
	Change from baseline, wk. 16	−4.2 (5.0)	−2.0 (7.2)	− 0.37
	Depression			
	Baseline	49.3 (7.9)	45.2 (6.0)	–
	Change from baseline, wk. 7	−3.7 (6.3)	0.3 (5.4)	−0.64
	Change from baseline, wk. 12	−3.8 (5.4)	1.6 (7.7)	− 0.78
	Change from baseline, wk. 16	−4.4 (7.0)	−0.6 (7.1)	−0.53
	Interest Sexual Activity			
	Baseline	43.0 (6.8)	43.8 (8.0)	–
	Change from baseline, wk. 7	−0.6 (5.8)	− 0.2 (4.6)	− 0.08
	Change from baseline, wk. 12	0.4 (4.9)	2.4 (4.2)	−0.43
	Change from baseline, wk. 16	0.7 (5.0)	−0.4 (6.9)	0.20
	Mental Health			
	Baseline	45.1 (5.1)	41.1 (5.0)	–
	Change from baseline, wk. 7	−5.0 (7.2)	− 5.3 (5.0)	0.05
	Change from baseline, wk. 12	−5.1 (5.0)	−4.8 (6.5)	−0.05
	Change from baseline, wk. 16	−5.2 (4.2)	−4.8 (5.0)	−0.11
	Orgasm			
	Baseline	2.9 (1.9)	3.0 (1.6)	–
	Change from baseline, wk. 7	0.0 (1.6)	0.6 (1.6)	−0.37
	Change from baseline, wk. 12	−0.2 (0.6)	0.1 (2.0)	−0.16
	Change from baseline, wk. 16	−0.3 (0.8)	0.2 (1.9)	−0.35
	Pain Intensity			
	Baseline	45.1 (8.6)	40.4 (9.0)	–
	Change from baseline, wk. 7	−1.6 (5.8)	0.6 (7.5)	−0.34
	Change from baseline, wk. 12	−2.4 (7.0)	0.6 (6.6)	−0.43
	Change from baseline, wk. 16	−3.2 (7.3)	0.8 (8.4)	−0.51
	Physical Health			
	Baseline	39.8 (3.1)	40.0 (4.4)	–
	Change from baseline, wk. 7	−6.3 (6.2)	−7.2 (6.8)	0.14
	Change from baseline, wk. 12	−6.6 (6.0)	−3.5 (5.7)	−0.52
	Change from baseline, wk. 16	−6.9 (7.3)	−6.9 (6.9)	0.01
	Satisfaction Sex Life			
	Baseline	49.1 (12.6)	46.7 (12.8)	–
	Change from baseline, wk. 7	−1.0 (10.4)	2.4 (6.0)	−0.38
	Change from baseline, wk. 12	−0.9 (6.6)	2.9 (12.6)	−0.41
	Change from baseline, wk. 16	−1.7 (8.2)	−0.3 (11.4)	−0.15
	Vaginal Discomfort			
	Baseline	48.0 (5.9)	48.0 (4.3)	–
	Change from baseline, wk. 7	0.7 (4.0)	−1.5 (3.8)	0.55
	Change from baseline, wk. 12	0.5 (4.1)	−1.5 (5.6)	0.43
	Change from baseline, wk. 16	−0.8 (3.2)	−2.3 (1.7)	0.51
	Sleep			
	Baseline	50.1 (6.2)	48.8 (6.7)	–
	Change from baseline, wk. 7	−2.3 (5.8)	0.2 (5.2)	−0.44
	Change from baseline, wk. 12	−2.5 (5.9)	1.0 (4.7)	−0.61
	Change from baseline, wk. 16	−3.8 (7.6)	−2.1 (5.0)	−0.26
	Quality of Life			
	Baseline (1–5)	3.1 (0.8)	2.6 (0.9)	–
	Change from baseline, wk. 7	−0.4 (0.6)	−0.4 (0.8)	0.10
	Change from baseline, wk. 12	−0.1 (0.5)	−0.3 (0.9)	0.34
	Change from baseline, wk. 16	−0.2 (0.5)	−0.3 (0.9)	0.09
MPN Symptom Assessment Form Outcomes	Fatigue			
	Baseline (0–10)	5.4 (2.3)	4.5 (2.8)	–
	Change from baseline, wk. 7	−0.7 (2.5)	−0.8 (3.1)	0.02
	Change from baseline, wk. 12	−0.5 (2.2)	−0.6 (2.3)	0.06
	Change from baseline, wk. 16	−0.7 (2.4)	0.6 (2.0)	−0.56
	Total Symptom Score			
	Baseline (0–100)	28.7 (15.6)	21.9 (13.5)	–
	Change from baseline, wk. 7	−1.4 (9.5)	−5.8 (11.2)	0.44
	Change from baseline, wk. 12	−2.7 (8.5)	−4.1 (9.5)	0.15
	Change from baseline, wk. 16	4.4 (8.8)	7.1 (8.7)	−0.31

Note: lower scores represent an improvement for each measured outcome. NIH PROMIS outcomes are converted to standardized t-scores

^a^cohen’s d was used for effect sizes

#### Effects of yoga on inflammatory biomarkers

There was a decrease in TNF-α from baseline to post-intervention in OLY participants (− 1.3 ± 1.5 pg/ml; ES = -0.87). Changes from baseline to post-intervention for IL-6 (ES = -0.26) were small.

## Discussion

This was the first study to explore the limited efficacy of a 12-week online yoga intervention among MPN patients on symptom burden and quality of life and (2) feasibility of remotely collecting inflammatory biomarkers that may be associated with disease, inflammation and symptom burden in a national MPN patient sample. Examination of *OLY* as opposed to in-person yoga may be a more sensible option not only in MPN patients, but also in other cancer patients due to its convenience, potential for dissemination for a rare disease with a dispersed population, and the barriers to in-person interventions that cancer patients report (i.e., fatigue, pain, transportation and scheduling conflicts) [25]. Additionally, many MPN patients often seek treatment from tertiary treatment centers that offer national and international consultative services due to the rarity of the disease where they would not be able to regularly attend in-person supportive care classes. MPN patients in the prior feasibility study conducted by Huberty et al. [16] reported the flexibility (i.e., the ability to do it at home whenever they want) to be the biggest benefit of participating in *OLY*.

### Yoga participation

Yoga participation assessed via Clicky averaged ~ 41 min/week whereas self-reported yoga participation averaged ~ 56 min/week. There was a significant difference between Clicky and self-reported yoga participation for all weeks except for week two (*p* = 0.11). The overall trend for both self-report and Clicky participation decreased as the study progressed.

Research suggests that physical activity is generally over-reported when compared to objectively-measured physical activity, with reasons including social desirability bias or a need for social approval [26, 27] However, it is also possible that self-report yoga minutes were higher if study participants recorded additional minutes spent outside of the prescribed yoga session videos (i.e., setting up for yoga session, cleaning up after yoga session, spending additional time in the “resting pose” at the end of a yoga session, etc.). Future studies should ask participants to comment on any additional time spent outside of the yoga class that they may have counted as “yoga minutes” on their self-report log to help in identifying any discrepencies between self-reported yoga and yoga as measured via Clicky.

### Patient-reported outcomes

There were small effects of the yoga intervention observed for sleep disturbances, pain intensity and anxiety. A moderate effect of the yoga intervention was observed for depression. These findings, in conjunction with the findings from a feasibility study conducted by Huberty et al. [16] in which 12 weeks of online yoga was feasible and illustrated improvements from pre-to-post intervention (no control group) in sleep disturbance and fatigue among MPN patients, lend further evidence to the preliminary effects of yoga on these outcomes. Therefore, future testing of the potential effects of online yoga among MPN patients is warranted.

Due to the pilot nature of this trial and a focus on preliminary effects and feasibility outcomes, we did not adjust for potential confounding factors. This approach has been recommended previously for feasibility and pilot trials to avoid placing an emphasis on hypothesis-driven outcomes that could be misinterpreted in feasibility and pilot studies that have based the sample size on power calculations [28]. The results herein provide information to motivate the design and execution, including observed effect sizes for all domains to assist with execution of future randomized controlled trials to determine efficacy in MPN patients.

### Blood draw feasibility and outcomes

The remote blood draw was feasible as ≥70% of study participants completed the measure at both time points Furthermore, a large effect size was observed for TNF-α (− 1.3 ± 1.5 pg/ml) from baseline to post-intervention. To the authors’ knowledge, there has not been a national feasibility study in cancer patients that assessed the collection of blood biomarkers remotely. One study that was conducted in a national sample of 591 inflammatory bowel disease (IBD) patients examined the feasibility of remote saliva collection and blood draws via: a) blood sample through a mobile phlebotomy service, b) blood sample through a local physician’s office, or c) saliva sample via a mailed saliva collection kit [29]. Of the 591 participants, 28.9% (*n* = 171) contributed a biospecimen, of which 90 were saliva samples, 47 were mobile phlebotomy blood samples, and 34 were physician’s office blood samples. Although this study was not conducted in cancer patients, they collected blood remotely in a national sample of patients. The blood draw completion rates of the present study were higher than that demonstrated in the IBD study, demonstrating the potential utility of this blood collection method in a national sample of MPN patients. This could be useful for future studies that intend to measure blood biomarkers in a national sample of patients and provides a potential methodological framework to follow.

Prior research indicates that increases in TNF-a are associated with a worsened symptom burden, a higher overall morbidity, and has been suggested as a potential driver of MPN disease progression [30]. Yoga has been shown to reduce inflammatory biomarkers in other cancer patients (particularly breast cancer) [31, 32]. Although the present study demonstrates a decrease of TNF-a from baseline to post-intervention, the number of participants completing the blood draw was small (*n* = 19/27) and there was no comparison to a control making these findings unreliable. However, the data warrants future research to test for the effects of online yoga on inflammation in MPN patients as compared to a control group.

### Limitations & lessons learned

There are some limitations to note in this pilot study that provided important learning opportunities to inform future research involving MPN patients:

1. Recruitment of a more representative sample of MPN patients – Our study was disproportionately biased towards females (i.e., 93.8%) and those that report regularly physical activity participation (i.e., 50% were ineligible in current study due to exceeding 150 min/week of moderate/vigorous-intensity physical activity). This is not representative of the general MPN patient population. Typical MPN patient population is ~ 53% female [33] and upwards of 61% of MPN patients report inactivity [2]. To eliminate bias, future studies could aim to recruit patients through both social media and clinics/hospitals. Physician referrals to interventions could potentially create a more diverse sample of participants. Furthermore, advertising the study as a “symptom management study” as opposed to a “yoga study” could further help to reduce an entirely self-selected study sample interested in yoga or already physically active.
2. Strategies for including patients with a broader range of depressive symptoms - A total of 37% of those ineligible for this study were ineligible due to reporting moderate-severe depression (according to PHQ-9 score ≥ 15). However, yoga may be beneficial for patients with moderate-severe depressive symptoms as yoga has been shown to improve depressive symptoms in other cancer patient populations [10–13] and cross-sectional work has identified a negative association between self-report yoga participation and reduced depressive symptoms in MPN patients [15]. Future studies should include a clinical psychologist/psychiatrist to oversee participants reporting moderate-severe depression (i.e., including them in the study population).
3. Identify potential confounding factors - Medications/treatments that may affect symptoms were not recorded throughout the study by participants, however, it is possible that study participants started or stopped taking medications, or received new treatments (unbalanced between groups) during their participation in the study. Future studies should have participants record their treatments and medications throughout the study to control for this potential confounding factor and to better determine effects of yoga on MPN patients.
4. Use alternatives to single-item self-report outcome measures – The fatigue and quality of life measures used in the present study were both single-item questions taken from validated measures. The fatigue measure was a single fatigue question from the MPN-SAF and the quality of life measure was a single question taken from the NIH PROMIS Global Health measure. It is possible that these single-item measures were not as sensitive to change when compared to multi-dimensional, comprehensive measures such as the Brief Fatigue Inventory (fatigue) [34–36] or the EORTC QLQ-C30 (quality of life) [37]. Future studies should use multi-diemsnional measures to assess outcomes.
5. Determine the effects of yoga on long-term outcomes - The present study included a 4-week follow-up period, however, longer follow-up is needed in future studies to determine the long-term effects of yoga participation. A recent systematic review and meta-analysis [13] of yoga randomized controlled trials in cancer patients showed that 4/10 of the included studies had a 12-week follow-up period. The other six studies did not include a follow-up period. Furthermore, none of these studies included MPN cancer patients. Future studies should include at least a 12-week follow-up period to fill a gap in the literature relating to the sustained effects of yoga on MPN patients.
6. Use behavior strategies to help participants achieve prescribed 60 min/week of yoga – Depsite prescribing a total of 60 min/week of yoga participation, participants averaged ~ 41 min/week as measured with Clicky. Future studies should employ techniques to increase compliance with the prescribed yoga minutes, including strategies that are based on the Health Belief Model that suggests cues to action (i.e., feedback) will stimulate behavior [38]. For example, research staff can provide study participants with text message or email reminders to participate in yoga when their weekly participation drops below a certain threshold. If participation remains low for multiple weeks in succession, research staff could then attempt to call the study participant to discuss a strategy to increase adherence to the yoga prescription. Additionally, the addition of a study participant group-based discussion forum may help to stimulate participation. The group forum would be a place for study participants to receive encouragement, ask questions, and express concerns. In our online yoga feasibility study [16], 82% (*n* = 23/28) of participants indicated that they would have been interested in participating in a group forum throughout the study (unpublished data).

## Conclusions

Despite major advances in the MPN treatment armamentarium over the last decade, those afflicted with the disease have persistent and often treatment-resistant symptoms. A 12-week OLY intervention demonstrated small effects on sleep disturbances, anxiety and pain intensity as well as moderate effects for depression in MPN patients. Our pilot data, although not enough to suggest online yoga as a novel treatment strategy for refractory disease-related symptoms, will inform future studies. Additionally, the remote collection of blood samples in a national sample of patients is feasible and provides a valuable option for researchers conducting nationwide studies in cancer patients. Patients participating in OLY revealed reduced TNF-α levels, however, these findings were from a small sample size and were not compared to a control group making them unreliable. The findings of this study will be used to inform the design of a future fully powered randomized controlled trial to determine efficacy of OLY in MPN patients and to clarify impacts on inflammation.

## Data Availability

The datasets used and/or analysed during the current study are available from the corresponding author on reasonable request.

## Abbreviations

CG: Control group
IBD: Inflammatory bowel disease
IL: Interleukin
MPN: Myeloproliferative neoplasm
MPN-SAF: Myeloproliferative neoplasm symptom assessment form
NIH PROMIS: National Institutes of Health patient reported outcomes measurement information system
OLY: Online yoga
TNF-a: Tumor necrosis factor-alpha
TSS: Total symptom score
